# Chemical Management Strategies of *Pimelea trichostachya* Lindl. Using Pre- and Post-Emergence Herbicides

**DOI:** 10.3390/plants13101342

**Published:** 2024-05-13

**Authors:** Rashid Saleem, Shane Campbell, Mary T. Fletcher, Sundaravelpandian Kalaipandian, Steve W. Adkins

**Affiliations:** 1 School of Agriculture and Food Sustainability, The University of Queensland, Gatton, QLD 4343, Australia; shane.campbell@uq.edu.au (S.C.); s.adkins@uq.edu.au (S.W.A.); 2 Queensland Alliance for Agriculture and Food Innovation, The University of Queensland, Coopers Plains, QLD 4108, Australia; mary.fletcher@uq.edu.au; 3 Department of Bioengineering, Saveetha Institute of Medical and Technical Sciences (SIMATS), Saveetha School of Engineering, Chennai 602105, Tamil Nadu, India

**Keywords:** *Pimelea trichostachya*, tebuthiuron, 2,4-D, metsulfuron-methyl, dry biomass, pasture

## Abstract

*Pimelea trichostachya* Lindl. is a native Australian forb responsible for livestock poisoning and reducing the productivity and sustainability of grazing enterprises. This study was conducted as a pot trial under controlled conditions to investigate an effective chemical management strategy for *P. trichostachya*, a method that did not leave standing dead plant material, as such material can also be toxic to grazing cattle. Three herbicides, including one pre-emergence (tebuthiuron) and two post-emergence herbicides (2,4-D and metsulfuron-methyl), were tested in pot trials for their efficacy on *P. trichostachya*. Results showed that tebuthiuron applied as either a granular (10% active ingredient, a.i.) or pelleted (20% a.i.) form efficiently reduced the emergence of *P. trichostachya* seedlings. Although some seedlings emerged, they perished within 7 days post treatment, leaving no residual plant matter. Testing now needs to be undertaken under field conditions to validate the findings within vegetation communities where potential non-target impacts need to be accounted for as well. The post-emergence application of 2,4-D and metsulfuron-methyl demonstrated that the highest efficacy and reduced application rates were achieved by treating earlier growth stages (i.e., seedlings) of *P. trichostachya* plants. In addition, the amount of toxic dead plant material was minimized due to the faster degradation of these small plants. These findings offer practical, cost-effective solutions for sustaining grazing lands from *P. trichostachya* challenges.

## 1. Introduction

The *Pimelea* genus Banks & Sol.ex Gaertn., encompassing small shrub and forb species commonly known as rice flowers, is indigenous to Australia, Lord Howe Island, New Zealand, and the island of Timor. A thorough exploration based on herbarium records and diverse botanical studies has unveiled a total of 140 distinct *Pimelea* species [1]. Among these, four species, *viz*., *P. simplex* F. Muell. subspecies simplex, *P. simplex* subspecies continua (J.M. Black) Threlfall, *P. trichostachya* (Lindl.), and *P. elongata* (Threlfell), stand out for their economic and animal welfare significance due to the presence of the toxin simplexin. It is a diterpenoid orthoester, and well known for its toxicity to cattle [2]. This toxicity holds historical importance, often referred to as Marree Disease or St. George Disease, and has been a persistent concern in the arid grazing regions of inland Australia [2]. *Pimelea trichostachya* tends to be the most widespread species. According to herbarium records and various regional botanical publications, it has been documented in New South Wales (NSW) [3], Queensland (QLD) [4,5], South Australia (SA) [6], Western Australia (WA), and the Northern Territory (NT) [7,8]. This plant demonstrates the ability to germinate and form seedlings under various environmental conditions, though it shows a preference for moderate temperatures, light, and mildly acidic soils [9]. All four poisonous species and subspecies are regarded as annuals [4]. They flower in spring and grow well in winter [10], usually suppressing their growth and further establishment during dry summers [11]. Management of *Pimelea* species can be particularly challenging for livestock producers in Australia, especially those who manage large grazing properties. The cost and logistics of chemical or physical management can be prohibitive for many producers when the infested area is extensive [12]. Therefore, exploring alternative management strategies is crucial to effectively control *Pimelea* species while avoiding unintended consequences.

According to a recent survey conducted in 2022 involving 32 producers, the average annual financial loss resulting from *Pimelea* poisoning has increased to AUD 67,000, and further findings revealed that chemical control and rotational grazing are the preferred methods employed for *Pimelea* management [13]. Hence, there arises a necessity for a novel and cohesive chemical control approach. However, implementing such a method poses challenges. This entails employing a post-emergence control method, utilizing cost-effective herbicides, selectively applied to emerging populations. Although significant field trials have been conducted by Silcock [14], a critical void still exists in the management paradigm of the use of innovative chemical control approaches, particularly those using pre-emergence herbicides. Further greenhouse studies are still needed to explore the use of herbicides to determine optimal application rates, evaluate efficacy, and identify the most appropriate timing for application. For the effective management of *Pimelea*, combining early-acting herbicides like tebuthiuron with post-emergence herbicides such as 2,4-dichlrophenoxyacetic acid (2,4-D) and metsulfuron-methyl may provide the best results. The key lies in executing this strategy early in the growth cycle, before significant vegetative material accumulates, mitigating the risk of toxic plant residue production. Currently, there is only one minor use permit issued by the Australian Pesticides and Veterinary Medicines Authority (Permit No. 13549) [15] for the limited use of glyphosate, metsulfuron-methyl, and 2,4-D for the management of *Pimelea* in pastures. Glyphosate, although included in the permit, is a non-selective herbicide and was not included in the current study, which sought to target *Pimelea* without damage to desirable vegetation. 

The herbicide 2,4-D, a member of the phenoxyacetic acid chemical group, is widely employed in agricultural systems to control a diverse range of broadleaf weeds and woody plants, including those in pastures and rangelands [16]. Renowned for its selectivity, efficiency, and cost-effectiveness, it is a popular choice in weed management [17] and importantly is registered for use on *Pimelea*. Metsulfuron-methyl, part of the sulfonylurea chemical group, serves as a common active ingredient in herbicide mixes targeting a broad spectrum of broad-leaved weeds. Its application can occur both before (pre-emergence) and after emergence (post-emergence) of the weeds [18]. Tebuthiuron, classified as a systemic herbicide in the thiadiazolyl ureas family, is utilized for controlling various herbaceous annuals, perennials, and woody weeds [19]. Employing post-emergence herbicide strategies may call for measures such as fencing or relocating cattle to clean areas until the toxic plant residues dissipate. Additionally, introducing the concept of nursery paddocks can be beneficial for the recovery of sick or vulnerable livestock. These smaller paddocks offer the advantage of providing fodder to the livestock, at the same time allowing for the treatment of any existing *Pimelea* invasion using pre-emergence herbicides. This targeted approach would ensure a more controlled and supportive environment for stock recovery. Notably, there has been a lack of research on the use of pre-emergence or early-acting herbicides for the chemical control of *P. trichostachya.*

The overarching goals of this pot trial study were twofold: firstly, to initiate a comprehensive trial involving early-acting herbicides, focusing on their efficacy and impact during the initial stages of plant development. To achieve this, the herbicide (tebuthiuron) was applied in various concentrations and formulations, as anecdotally it is one that landholders in the affected areas are familiar with and use for other purposes (e.g., woody weed control). Secondly, we aimed to conduct a post-emergence herbicide trial, exploring the effectiveness of herbicides applied after the emergence of plants. This entailed building on previous research [14] and optimizing the application of 2,4-D and metsulfuron-methyl. These herbicides were tested individually and at different rates, with applications targeted at various growth stages of *P. trichostachya* (i.e., seedling, vegetative, pre-flowering). Both trials were crucial components of the research initiative and aimed to unveil the potential benefits and limitations of early-acting herbicides while scrutinizing the performance of post-emergence herbicides for sustainable, long-term control. The findings of this research will contribute to the development of enhanced management strategies to prevent or control *P. trichostachya* influxes. 

## 2. Results

### 2.1. Effect of Pre-Emergent Tebuthiuron Applications (Experiment 1)

**Scheme 1:**
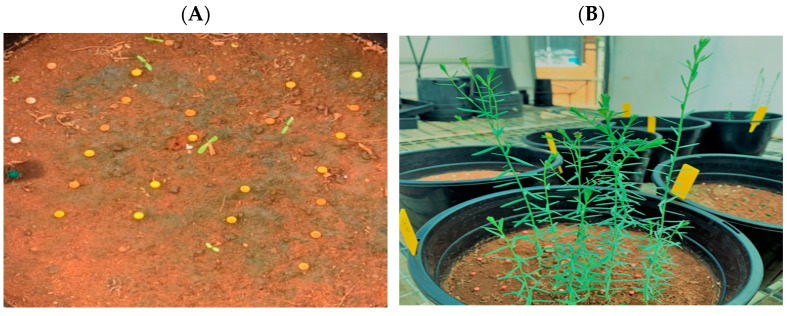
(**A**) A small number of seedlings were observed emerging after the application of either pelleted or granular tebuthiuron, but all died within 7 days after having attained a maximum of ca. 5 mm in height. (**B**) Healthy plants in control pots (no herbicide application) after 56 days of application.

**Figure 1:**
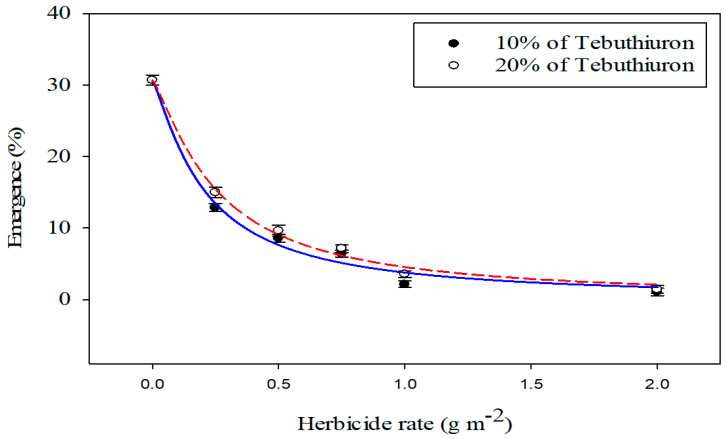
Cumulative rate–response curves for the emergence of *P. trichostachya* seedlings using tebuthiuron granules (10% a.i. g m^−2^) (-●-) and pellets (20% a.i.; g m^−2^) (-o-) measured after 56 days from pooled data of repeated experiments using scarified (1.15 mM for 24 h) seeds pre-soaked in GA_3_. In each of the two experiments, there were 50 seeds per treatment with four replicates. The data have been corrected due to having only 70% filled seed, and the bars indicate standard errors of the plotted means.

The interaction between tebuthiuron formulation and application rates significantly influenced the emergence of *P. trichostachya* seedlings (*p* < 0.05; Figure 1). Both formulations exhibited an inverse correlation between emergence and application rate (Figure 1 and Table S1). Generally, the 10% active ingredient (a.i.) granular formulation showed an insignificant advantage in curtailing the emergence of *P. trichostachya* as compared to the 20% a.i. pelleted formulation, particularly at the lower application rates. In the untreated control group, the average emergence was 31%, while at the lowest rate of 0.25 g a.i. m^−2^, the emergence was 13 and 15% for the 10 and 20% a.i. formulations, respectively. Initially, there were also more seedlings emerging from the lower doses as compared to higher doses. This pattern persisted with increasing application rates, giving the lowest seedling emergence rate at the highest herbicide rate (2 g a.i. m^−2^) of the 10% formulation (1.0%) (Figure 1). The Effective Dose (ED_50_) values, representing the dose required for 50% inhibition of seedling emergence, were 0.20 g a.i. m^−2^ for the 10% formulation and 0.25 g a.i. m^−2^ for the 20% formulation. Nevertheless, both the 10% granular and 20% pellet formulations, across all application rates, effectively eliminated all seedlings within one week of emergence (Scheme 1A). In contrast, the untreated control maintained live seedlings (5) even at 56 days after application (DAAs) (Scheme 1B).

### 2.2. Effect of Post-Emergence Herbicides (Experiment 2)

**Figure 2:**
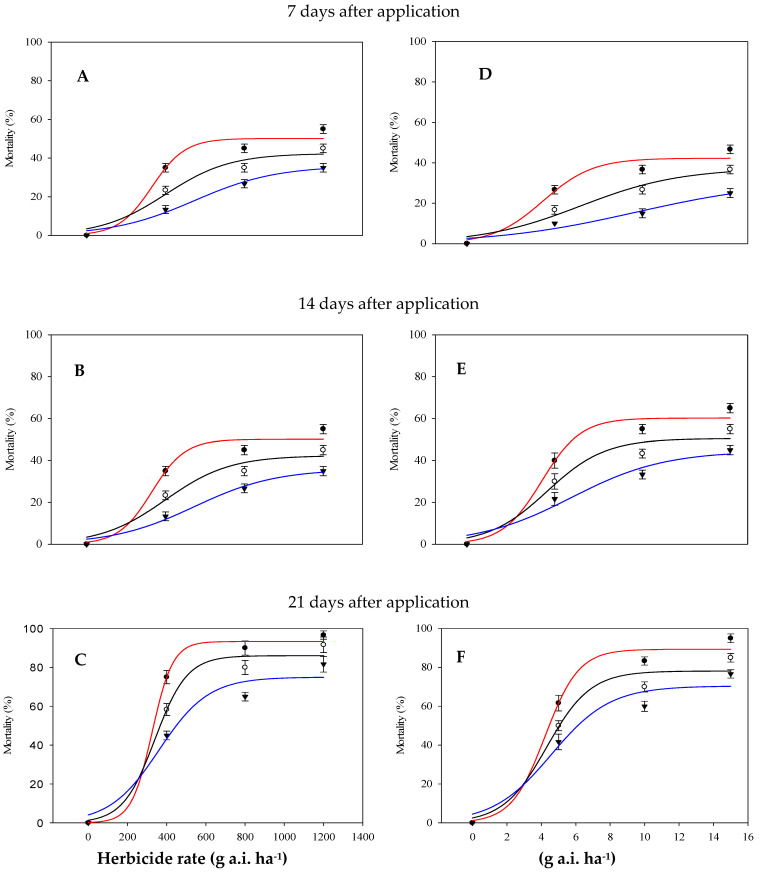
Rate–response curves for herbicide-generated mortality of *P. trichostachya* at three growth stages (seedling (-●-), vegetative (-o-), pre-flowering (-▼-)) and measured 7 (**A**), 14 (**B**), and 21 (**C**) days after application in response to 2,4-D. Rate–response curves for herbicide-generated mortality of *P. trichostachya* at three growth stages (seedling (red), vegetative (black), pre-flowering (blue)) taken 7 (**D**), 14 (**E**), and 21 (**F**) days after application in response to metsulfuron-methyl. Error bars represent ± two standard errors.

Both herbicides significantly influenced the mortality rates of *P. trichostahya* (*p* < 0.05). The herbicide mortality rates were found to be higher at the seedling stage, e.g., at 7, 14, and 21 DAAs (Figure 2, Table S2). Additionally, higher application rates of both herbicides led to progressively increased mortality rates until 21 DAAs. Notably, the level of mortality varied between the two herbicides across all application rates. Comparatively, 2,4-D displayed greater efficacy in controlling *P. trichostachya* when compared to metsulfuron-methyl (Figure 2). 

At 21 DAAs, the ED_50_ value for 2,4-D applied at the seedling stage was 300 g a.i. ha^−1^, representing the dose required to cause 50% mortality. At a CI of 95%, the application rate ranged from 200.3 to 450.4 g a.i. ha^−1^. At the vegetative stage, the ED_50_ value increased to 310 g a.i. ha^−1^, with a range of 308.9 to 370.8 g a.i. ha^−1^. At the pre-flowering stage, the ED_50_ value was 339 g a.i. ha^−1^, and the rate range spanned from 337.4 to 384.3 g a.i. ha^−1^. 

Similarly, different levels of sensitivity among growth stages and rates in response to applications of metsulfuron-methyl were observed at all assessment times (Figure 2D–F, Table S3). At 21 DAAs, the ED_50_ value at the seedling stage for metsulfuron-methyl was 4.2 g a.i. ha^−1^, ranging from 3.8 to 4.5 g a.i. ha^−1^ at a CI of 95%. Similarly, during the vegetative stage, the ED_50_ value was 4.3 g a.i. ha^−1^, ranging from 4.0 to 4.5 g a.i. ha^−1^. At the pre-flowering stage of *P. trichostachya,* the ED_50_ value increased to 4.5 and ranged from 4.2 to 4.9 g a.i. ha^−1^. Next, the tolerance factor (TF) was measured, which is a measure of how much the response (efficacy) differs between two different conditions, often representing different growth stages in this study. When comparing the TF between the herbicides 2,4-D and metsulfuron-methyl across different growth stages, it is obvious that plants exhibited a greater tolerance to 2,4-D (Tables S2 and S3). At 21 DAAs, at the seedling stage, the TF for 2,4-D was 1.30, which indicates a moderate level of tolerance, while for metsulfuron-methyl, the TF was just 0.42, suggesting a significantly lower tolerance. As the plants progressed to the vegetative stage, the TF for 2,4-D increased to 1.35, reflecting a slightly higher tolerance, while the TF for metsulfuron-methyl was 0.43, indicating a marginal increase, but this was still significantly lower than for 2,4-D. The trend continued at the flowering stage, with a TF of 1.44 for 2,4-D, representing the highest level of tolerance, whereas the TF for metsulfuron-methyl was only 0.45, showing a smaller increase in tolerance. Overall, this comparison demonstrates that plants have a much greater tolerance to 2,4-D compared to metsulforan-methyl at all growth stages, with the most pronounced differences occurring at the seedling and vegetative stages.

**Figure 3:**
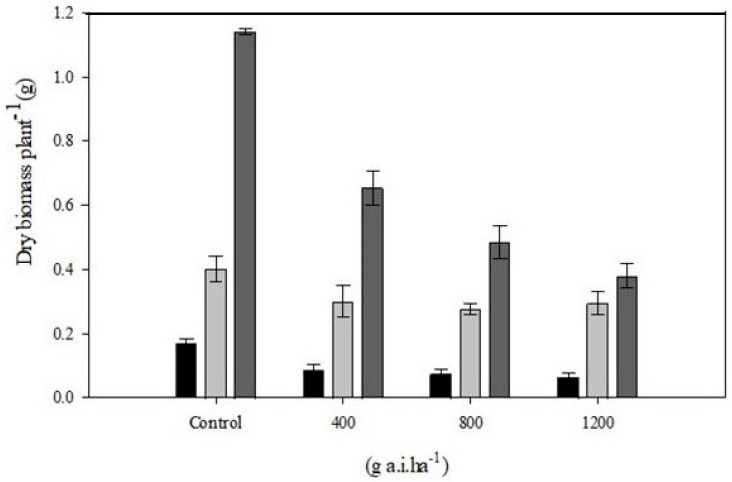
The cumulative impact of post-emergence 2,4-D application on the dry biomass (g plant^−1^) of *P. trichostachya* was examined using combined data from repeated experiments with varying doses (0, 400, 800, or 1200 g a.i. ha^−1^). Growth stages in *P. trichostachya* are denoted by a color-coded legend: a black bar signifies the seedling stage, light grey denotes the vegetative phase, and dark grey indicates the mature stage. Error bars are included to represent ± two standard errors of the means.

**Figure 4:**
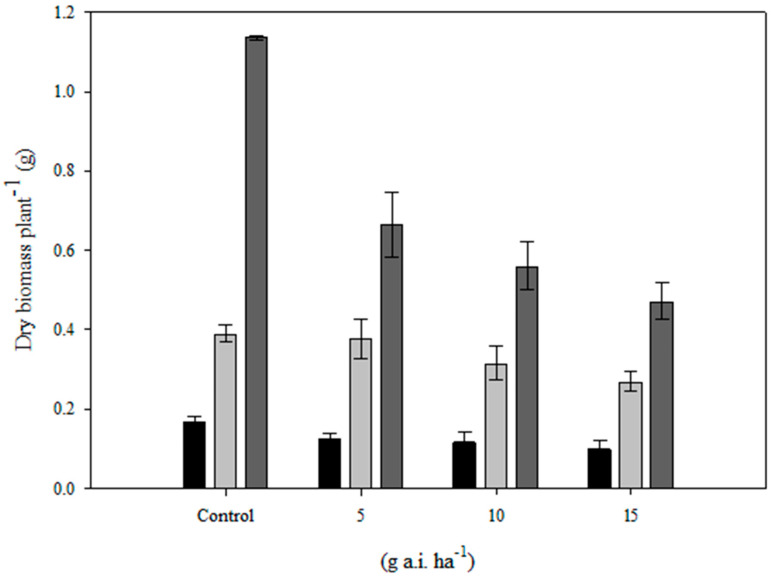
The cumulative impact of post-emergence metsulfuron-methyl application on the dry biomass (g plant^−1^) of *P. trichostachya* was examined using combined data from repeated experiments with varying doses (0, 5, 10, or 15 g a.i. ha^−1^). Growth stages in *P. trichostachya* are denoted by a color-coded legend: a black bar signifies the seedling stage, light grey denotes the vegetative phase, and dark grey indicates the pre-flowering stage. Error bars are included to represent ± two standard errors of the means.

Significant differences (*p* < 0.05) were observed between herbicides and between growth stages in the amount of *P. trichostachya* biomass remaining at 21 DAA (Figure 3 and Figure 4). Metsulfuron-methyl treatments retained the highest dry biomass compared to 2,4-D treatments. In terms of growth stages, dry biomass increased with increasing plant maturity. For the herbicide treatments, the maximum dry biomass of *P. trichostachya* occurred when the lowest rate of metsulfuron-methyl (5 g a.i. ha^−1^) was applied at the pre-flowering stage (Figure 4). 

## 3. Discussion

This study has highlighted the potential of tebuthiuron to provide pre-emergence control of *P. trichostachya,* with both small granular and larger pelleted formulations giving effective control of seedling emergence at comparable rates in a pot-based trial. Testing now needs to be undertaken under field conditions to validate the findings within vegetation communities where potential non-target impacts need to be accounted for as well as the environmental footprint of these herbicides. A post-emergence trial confirmed the efficacy of 2.4-D amine and metsulfuron-methyl in three growth stages (i.e., seedlings, vegetative, pre-flowering) of *P. trichostachya*, with herbicide applications at the seedling stage proving most successful in terms of mortality and reducing residual biomass that, if eaten, could poison livestock. 

### 3.1. Efficacy of Tebuthiuron in Managing P. trichostachya

**Scheme 2:**
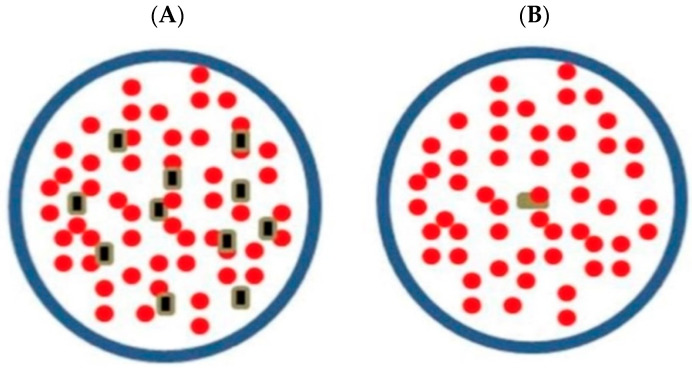
The pattern of the soil surface application of tebuthiuron in the two experimental procedures. Tebuthiuron was applied as (**A**) experimental tebuthiuron granules 10% a.i. (black squares) and (**B**) as Graslan pellets 20% a.i. (single brown square) to pots each containing 50 seeds (red dots) planted 2 mm deep under the soil surface.

The 10% a.i. granular and 20% a.i. pelleted products exhibited similar efficacies in reducing the emergence of *P. trichostachya* seedlings when compared at equivalent levels of the active ingredient (Figure 1; Scheme 2A,B). However, the experimental granular tebuthiuron formulation showed significantly better results at lower application rates. This inconsistency in effectiveness could potentially be attributed to the size of the respective products. The smaller granules of the experimental tebuthiuron formulation were able to be evenly spread over the soil surface of the pots (Scheme 1A), increasing the chances of contact between the herbicide and germinating seeds. On the other hand, in the pots allocated the 20% pelleted formulation, only a single larger pellet was placed in the centre (Scheme 1B), potentially resulting in some germinating seeds not coming into direct contact with the herbicide. As a result, these seeds could develop into seedlings. However, it is important to note that despite the emergence of any seedlings, they eventually succumbed within 7 days, while still in the early cotyledonary growth stage. This indicates that even if some seeds managed to escape direct contact with the herbicide, their growth and development were hindered, leading to their subsequent mortality. 

Furthermore, it is worth noting that the early seedlings of *P. trichostachya* decayed rapidly without leaving any toxic residues on the soil surface. This is particularly important to ensure the safety of animals that may accidentally consume the dead stem material, as the toxin remaining in dead stalks can have detrimental health effects, including severe illness and even death [2]. While the results from the pot trial are promising, it is crucial to validate the efficacy of tebuthiuron in a field trial conducted in a *P. trichostachya*-infested paddock. The use of commercially available tebuthiuron pellets should be sufficient for testing, but consideration could also be given to include a recently released liquid tebuthiuron formulation. This liquid formulation may provide more even coverage and potentially improved efficacy, especially at lower application rates. Additionally, the field trials should be carried out over an extended period to determine the duration of residual control provided by different rates of tebuthiuron against *P. trichostachya*. While the current pot trial demonstrated good residual control for 56 days, it is important to consider that the seedbank in the field will consist of seeds of varying ages and levels of dormancy. Previous studies have indicated that *P. trichostachya* seeds can remain viable in the seedbank for more than 2 years, posing a potential threat for future outbreaks [20]. Therefore, a longer-term assessment is necessary to fully understand the long-lasting effects of different tebuthiuron rates on *P. trichostachya* seedbank dynamics and control. 

During an extended field trial, it is expected that a combination of germinable and dormant seeds will be present in the *P. trichostachya* seedbank. The germinable seeds will remain dormant until favourable conditions, such as rainfall exceeding 25 mm, trigger their germination, as was reported for another herbaceous plant, florestina (*Florestina tripteris*) [21]. Like the research conducted on florestina, it would be beneficial to replicate trials to identify residual herbicides that can effectively prevent Pimelea seedling regrowth for an extended period. This would provide valuable insights into the duration of tebuthiuron’s control over *P. trichostachya.* However, caution should be exercised when considering the use of tebuthiuron beyond the current pot trial, as it can adversely affect native tree species, including Eucalyptus [22]. As a result, the suitability of tebuthiuron may be more appropriate for use within confined areas, such as hospital paddocks, where potential impacts on non-targeted vegetation can be minimized.

### 3.2. Efficacy of Post-Emergence Herbicides for Managing P. trichostachya

This study established the effectiveness of both 2,4-D and metsulfuron-methyl herbicides in controlling *P. trichostachya* across all three growth stages (Figure 2). Notably, applying these herbicides during the seedling stage proved crucial in minimizing residual biomass, a key factor in reducing the risk of Pimelea poisoning in livestock. Additionally, metsulfuron-methyl demonstrated pre-emergent effects on other Asteraceae weeds like *Florestina tripteris D.C. 1836* at 18 g a.i. ha^−1^ [21] and *Parthenium hysterophorus* in the field [23]. Goodall and Erasmus [22] recommended applying metsulfuron-methyl to *Chromolaena odorata* L. regrowth of slashed plants under 1.5 m at 15 g a.i. per 100 L. The timing of herbicide application, particularly in the cooler months, is crucial, contingent upon prevailing weather conditions and rainfall [20]. Considering economic factors, the application of herbicides on large grazing properties (>10,000 ha) appears uneconomical. A more focused approach on strategic areas like watering points, where plants can be easily targeted, is recommended [14,20]. Herbicide application is advised during early developmental stages, especially in hospital paddocks.

## 4. Materials and Methods

### 4.1. Experiment 1 (Early-Acting Herbicide Trial)

This experiment was undertaken in a greenhouse at The University of Queensland Gatton Campus during 2020–2021 and investigated the effect of pre-emergence herbicide treatments of tebuthiuron to prevent *P. trichostachya* seedling emergence. This was a pot-based experiment using a red sandy soil collected from the 0 to 30 cm layer of a *P. trichostachya*-affected paddock located in the Maranoa Shire (−27.3860 S 148.4229 E). The soil collected from a *P. trichostachya*-infested field was used to simulate real-world soil conditions in a controlled glasshouse situation. After collection, the soil was sun-dried for a period of 7 days and then crushed and sieved through a 2 mm mesh. It should be noted that despite its origin, preliminary studies confirmed that the soil did not contain any viable *P. trichostachya* seeds capable of germination.

Single-seeded fruit (hereafter referred to as seeds) for the experiment were collected at the above site from mature *P. trichostachya* plants growing in a paddock where oats (*Avena sativa* L.) had been previously planted. Subsamples of the seeds (50 seeds) were subjected to X-ray examination to assess the seed fill percentage, which was found to be 70% filled. Filled seed refers to those seeds that have reached maturity and are fully developed within the seed coat and which are identifiable through X-ray. To overcome physical dormancy, the seeds were scarified according to the established method previously described [12]. Additionally, a pre-soaking treatment was applied using GA_3_ (gibberellic acid) at a concentration of 1.15 mM for a duration of 24 h. This pre-soaking treatment was applied to help overcome any physiological dormancy present in the seeds. By carefully preparing the seeds for the experiment, ensuring their viability, and removing any dormancy barriers, the subsequent analysis of pre-emergence herbicide treatments can provide valuable insights into their effectiveness in preventing *P. trichostachya* seedling emergence.

A total of 8 kg of the prepared soil was allocated to each of the 96 black plastic pots, with each pot having a 25 cm diameter. Next, a small quantity of soil was carefully removed from the surface of each pot, and 50 pre-treated *P. trichostachya* seeds (as described earlier) were placed onto the exposed soil surface in each pot. The removed soil was then gently applied back to cover the seeds, ensuring they were buried at a depth of approximately 2 mm. Finally, a light spray through a sprinkler irrigation system was provided to ensure adequate moisture for seed germination. By following these procedures, the experiment aimed to simulate natural conditions and provide a suitable environment for the germination and growth of *P. trichostachya* seedlings.

The herbicide treatments used included two formulations of tebuthiuron. The first was a commercially available product (Graslan^®^ pellets) that contained 200 g a.i. kg^−1^ (20% a.i.). The second was an experimental formulation (supplied by the Queensland Department of Agriculture and Fisheries) consisting of small granules that contained 100 g a.i. kg^−1^ (10% a.i.). Prior to experimentation, chemical analysis undertaken at the Queensland Department of Agriculture and Fisheries’ Chemical Residue Laboratory (Coopers Plains, Brisbane) confirmed that the reported quantity of active ingredient was accurate for both pellet and granular products. In the experiment, both the granular and pelleted formulations of the herbicide were applied at five different rates: 500, 1000, 1500, 2000, and 4000 g of active ingredient per hectare (g a.i. ha^−1^). To ensure accurate comparisons, a non-treated control group was also included. It is important to note that the granular formulation had a lower concentration of tebuthiuron compared to the pelleted formulation. Therefore, to achieve an equivalent level of active ingredient for each rate, the granular formulation had to be applied at double the quantity of product compared to the pelleted formulation. The specific details of the rates and application quantities can be found in Table 1. 

The experimental design aimed to investigate whether the size of the herbicide product, either in the form of larger pellets or smaller granules, had any impact on its efficacy. Specifically, the study sought to determine if there were any differences in effectiveness when using a lower amount of the larger, more concentrated pellets versus a higher amount of the smaller, less concentrated granules. To ensure accuracy and consistency, a single pellet, appropriate in weight for the respective rate, was placed at the centre of each pot designated for pellet treatments. On the other hand, for the granular formulation treatments, the granules were evenly spread over the soil surface by hand in a random pattern (as shown in Scheme 2). The trial was conducted over a period of 56 days, during which the emergence of *P. trichostachya* seedlings was monitored based on their surface appearance. 

### 4.2. Experiment 2 (Post-Emergence Herbicides)

This experiment was conducted at The University of Queensland Gatton Campus during 2020–2021. For the post-emergence herbicide trial, a soil mixture was prepared by combining 1 kg of soil with 0.75 L of sterilized Gatton media (Osmocote 8–9M, Osmocote 3–4M, Nutricote 7M, containing coated iron, a moisture aid, dolomite, and Osmoform). This soil mixture provided the optimal growth medium for the plants. Subsequently, 1.75 kg of the soil mix was placed into each black plastic pot with a diameter of 15 cm. This ensured that each pot had sufficient soil volume to support the growth of the *P. trichostachya* plants during the trial.

To ensure consistent growth stages of *P. trichostachya* plants for herbicide application, the seed lots underwent pre-treatment to overcome physical and physiological dormancy. Following pre-treatment, the seeds were planted into individual Jiffy pellets (Jiffy Products International, AS, Norway), with a consistent 15-day interval between each planting. This strategic planting approach was implemented to promote the development of *P. trichostachya* plants at three distinct growth stages, facilitating subsequent herbicide applications: first, when the plants were 45 days old, marked by the presence of 32 leaves per plant; second, at 30 days old, corresponding to the 26-leaf stage; and finally, at 15 days old, characterized by the 12-leaf stage. Consequently, based on their growth stages, plants were classified as pre-flowering, vegetative, or seedlings. 

The pots were then placed inside a poly-tunnel located at the Nursery at The University of Queensland (UQ) Gatton Campus. To assess the efficacy of the herbicides, specifically 2,4-D and metsulfuron-methyl, different treatment combinations were employed. The herbicides were applied at varying rates: 0, 400, 800, and 1200 g a.i. ha^−1^ for 2,4-D and 0, 5, 10, and 15 g a.i. ha^−1^ for metsulfuron-methyl. To perform the herbicide application, the plants were relocated from the poly-tunnel to the pesticide application facility at UQ Gatton. Environmental conditions at the time of herbicide application comprised an air temperature of 25 ± 2 °C and a relative humidity of 65 ± 5%. Herbicide solutions were prepared in distilled water and applied 50 cm above the top of the seedlings using a track sprayer fitted with a Teejet nozzle (8003E), delivering 100.38 L ha^−1^ at 300 kPa. All applied herbicide application treatments included a bio-degradable wetting and spreading surfactant (BS1000; Nufarm Australia Limited ACN 004 377 780 103-105 Pipe Road, Laverton North Victoria 3026) at 0.1% (*v*/*v*). Plant mortality was recorded at 7, 14, and 21 DAAs, and dry weight was recorded 21 days after herbicide treatment [24].

### 4.3. Mortality Assessment

The damage caused by the post-emergence herbicides (2,4-D and metsulfuron-methyl) was visually assessed, with their visual appearance recorded (e.g., tissue colour and necrosis, and other aberrations; see Table 2) at 7 DAAs, 14 DAAs, and 21 DAAs [24] by using a modified scale suggested by Frans [25], on which 0 represents no herbicide damage to the plant and 100 represents death after herbicide application (Table 2; [26]). At 21 DAAs, shoots above the soil surface were cut and placed in an oven for 72 h at ca. 65 °C, and afterwards the dry biomass of *P. trichostachya* was determined. 

### 4.4. Experimental Design and Statistical Analyses

Visual injury (%) over time was regressed over herbicide treatments using the three-parameter sigmoid–sigmoidal model; the three-parameter logistic model is commonly expressed as

$$y=\frac{a}{1+{e^{-}}^{\left(\frac{x-x_{0}}{b}\right)}}$$

In this regression model, *b* is the slope, *a* is the maximum visually assessed control, x is the herbicide rate (g a.i. ha^−1^), and *x*_0_ is the rate providing 50% response of the variable. Analysis of the rate–response curves was performed separately for *P. trichotachya* plants and their growth stages, and ED_50_ was estimated through Sigmaplot 14.5. Finally, the tolerance factor (TF) was calculated through the quotients of ED_50_ of *P. trichotachya* by ED_50_ and of the recommended rate of the herbicide. The tolerance factor (TF) is a measure of how much the response (efficacy) differs between two different conditions, often representing different growth stages, as in this case. This ratio gives a quantitative measure of the difference in sensitivity or tolerance between the two conditions. If TF > 1, it indicates that the plant is more tolerant at the second growth stage compared to the first. If TF < 1, it indicates greater sensitivity at the second condition.

The pre-emergence experiment was arranged in a completely randomized design giving equal importance to all the treatment factors, and repeated. Each experimental treatment consisted of four replicate pots, each containing 50 seeds. There were three replications in the post-emergence trial, which was also arranged in a completely randomized design and repeated. The significance of means of individual treatment factors and their interactions was estimated through ANOVA in all experiments. Means were separated using Fisher’s protected LSD test at *p* = 0.05. Thus, the treatment means were presented in bar charts with ±SE of means using Sigma Plot v. 14.5. 

## 5. Conclusions

The herbicides tested on *P. trichostachya* in two separate experiments displayed effectiveness in different ways: tebuthiuron prevented seedling recruitment, while 2,4-D and metsulfuron-methyl controlled post-emergence growth at different developmental stages. Regarding the prevention of seedling emergence, both formulations of tebuthiuron (10% a.i. granular and 20% a.i. pelleted) exhibited similar effectiveness, with the experimental granular tebuthiuron (10%) demonstrating significantly higher efficacy at lower concentrations, and this pre-emergence herbicide is recommended for smaller nursery paddocks. Nonetheless, even at lower doses, any seedlings that did manage to emerge perished within 7 days during the early cotyledonary growth stage, ensuring the absence of any lingering toxic plant residues on the soil surface. Post-emergence treatments resulted in notable mortality and a significant decrease in the dry matter of *P. trichostachya* when compared to the untreated control. Both post-emergence herbicides are recommended for main grazing paddocks as they demonstrated a gradual lethal effect on *P. trichostachya* plants. However, it is important to note that the residual dead stalks produced with the use of post-emergence herbicides might retain toxicity. Therefore, it is recommended that such herbicides be utilized only during the early stages of *P. trichostachya* development. 

Field trials in *P. trichostachya*-infested areas are recommended to explore alternative tebuthiuron formulations for improved efficacy. It is noted that relying solely on herbicides may be economically impractical for large properties, suggesting a need for integrated management approaches. Beyond chemical control, complementary management practices, including strategic grazing, pasture renovation with competitive plant species, and vigilant monitoring for early infestation detection, play a pivotal role in mitigating the impact of *P. trichostachya* on pastures and livestock. This integrated approach offers a comprehensive strategy to address the challenges posed by *P. trichostachya* in agricultural settings.

## Figures and Tables

**Table 1:** Rates of the commercial (i.e., Graslan pellets (20% a.i.) and experimental tebuthiuron granules (10% a.i.) on a per ha and per pot basis utilized in treatments T1 to T6 to supply equivalent tebuthiuron doses.

	Graslan Pellets (g ha^−1^)	Experimental Granules (g ha^−1^)	Tebuthiuron (g a.i. ha^−1^)	Graslan Pellets (g pot^−1^)	Experimental Granules (g pot^−1^)
T1 ^1^	0	0	0	0.000	0.000
T2	2500	5000	500	0.012	0.024
T3	5000	10,000	1000	0.024	0.049
T4	7500	15,000	1500	0.036	0.073
T5	10,000	20,000	2000	0.049	0.098
T6	20,000	40,000	4000	0.098	0.196

^1^ Untreated control.

**Table 2:** Herbicide visual mortality rating system based on the morphological symptoms that appeared on the foliage of plants and the extent of the damage caused by herbicides.

Mortality Rating System (%)	Morphological Responses
0	No visible symptoms
10	Negligible discolouration, distortion, and/or stunting barely seen
20	Slight damage discolouration, distortion, and or stunting clearly seen
30	Moderate damage: moderate discolouration, marked distortion, and/or stunting. Recovery expected
40	Substantial damage: much discolouration, distortion, and stunting. Some damage probably irreversible
50	Majority of plant tissue is damaged, mostly irreversibly: substantial necrosis; discolouration, and distortion
60	Nearly all the plant tissue damaged, mostly irreversibly, with some plants killed (<40%): substantial necrosis and distortion
70	Severe damage, necrosis, and wilting
80	Very severe damage, substantial necrosis, and wilting
90	Extreme irreversible damage with <10% green tissue visible, most tissue discoloured and distorted permanently or desiccated
100	Complete loss of plant

## Data Availability

Data are contained within the article.

## Supplementary Materials

The following supporting information can be downloaded at: https://www.mdpi.com/article/10.3390/plants13101342/s1, Table S1. Equations describing the seedling emergence inhibition and the ED_50_ values of *P. trichostachya*, measured 56 days after treatment in response to tebuthiuron granules (10%) and pellets (20%) applied at various rates. R^2^ is the coefficient of determination. Table S2. Equations describing the 2,4-D-induced mortality with 95% confidence intervals (CIs), the tolerance factor (TF), and ED_50_ values for three growth stages, measured at 7, 14, and 21 days after application (DAA). Table S3. Equations describing the metsulfuron-methyl-induced mortality with 95% confidence intervals (CIs), the tolerance factor (TF), and ED_50_ values for three growth stages, measured at 7, 14, and 21 days after application (DAAs).
