# Supplementary material for: Chemical Management Strategies of Pimelea trichostachya Lindl. Using Pre- and Post-Emergence Herbicides
Source: Plants (Basel). 2024 May 13;13(10):1342. doi: 10.3390/plants13101342 (PMC11125264; doi:10.3390/plants13101342)
Supplement: Supplementary file 1 [file plants-13-01342-s001.zip › plants-3012623-supplementary.pdf]

Supplementary Data

Table S1. Equations describing the seedling emergence inhibition and the ED_{50} values of *P. trichostachya*, measured 56 days after treatment in response to tebuthiuron granules (10%) and pellets (20%) applied at various rates. R^2 is coefficient of determination.

**Herbicides**	**Equations**	**R^2**	**ED_{50}***	
	f = a/(1+exp (-(x-0.20)/b))		g a.i. m^{-2}	CI 95%
Experimental granules (10% a.i.)	f = 30.67/(1+exp (-(x-0.20)/1.23))	0.96	0.20	0.18-0.21
Graslan pellets (20% a.i.)	f = 30.66/(1+exp (-(x-0.25)/1.27))	0.96	0.25	0.23-0.26

* Sigmoidal equation of three parameters; a and b are regression parameters related to intercept and slope on a logit scale; ED_{50} is the tebuthiuron rate (g a.i. m^{-2}) required to cause 50% reduction in emergence.

Table S2. Equations describing the 2,4-D-induced mortality with 95% confidence intervals (CI), the tolerance factor (TF) and ED_{50} values for three growth stages, measured at 7-, 14-, and 21-days after application (DAA).

Growth stages	Equations	R^2	ED_{50} (a.i. ha^{-1})	CI 95% (a.i. ha^{-1})	TF
			Mortality at 7-DAA		
Seedling	f = 50.15/(1+exp(-(x-330.77)/ 84.40))	0.92	355	295.2 - 366.3	1.32
Vegetative	f = 42.36/(1+exp(-(x-398.84)/ 163.25))	0.95	340	362.0 - 435.6	1.59
Pre flowering	f = 35.90/(1+exp(-(x-545.81)/ 207.08))	0.94	369	489.1 - 602.4	2.18
			Mortality at 14-DAA		
Seedling	f = 68.36/(1+exp(-(x-320.92)/ 62.53))	0.98	315	255.5 - 386.2	1.28
Vegetative	f = 56.89/(1+exp(-(x-337.63)/ 88.22))	0.98	332	309.9 - 365.3	1.35
Pre flowering	f = 45.31/(1+exp(-(x-352.36)/ 97.87))	0.92	376	324.5 -3 80.1	1.40
			Mortality at 21-DAA		
Seedling	f = 93.34/(1+exp(-(x-325.42)/ 52.96))	0.99	300	200.3 - 450.4	1.30

Vegetative	f = 86.05/(1+exp(-(x-339.88)/ 81.88))	0.97	310	308.9 - 370.8	1.35
Pre flowering	f = 75.00/(1+exp(-(x-360.92)/ 126.11))	0.98	338	337.4 - 384.3	1.44

Table S3. Equations describing the metsulfuron-methyl-induced mortality with 95% confidence intervals (CI), the tolerance factor (TF) and ED_{50} values for three growth stages, measured at 7-, 14-, and 21-days after application (DAA).

Growth stages	Equations	R^2	ED_{50} (a.i. ha^{-1})	CI 95% (a.i. ha^{-1})	TF
			Mortality at 7-DAA		
Seedling	f = 42.28/(1+exp(-(x- 4.33)/ 1.42))	0.91	4.3	3.9 - 4.6	0.43
Vegetative	f = 37.32/(1+exp(-(x- 6.47)/ 2.84))	0.88	6.4	5.6 - 7.2	0.64
Pre flowering	f = 31.20/(1+exp(-(x- 9.65)/ 4.03))	0.82	9.6	7.1 - 12.0	0.96
			Mortality at 14-DAA		
Seedling	f = 60.26/(1+exp(-(x- 4.25)/ 1.12))	0.93	4.2	3.8 - 4.6	0.42
Vegetative	f = 50.54/(1+exp(-(x- 4.56)/ 1.65))	0.90	4.5	4.1 - 4.9	0.45
Pre flowering	f = 44.59/(1+exp(-(x- 5.94)/ 2.65))	0.88	5.9	5.2 - 6.6	0.59
			Mortality at 21-DAA		
Seedling	f = 89.34/(1+exp(-(x- 4.22)/ 0.98))	0.96	4.2	3.8 - 4.5	0.42
Vegetative	f = 78.15/(1+exp(-(x- 4.31)/ 1.26))	0.95	4.3	4.0 - 4.5	0.43
Pre flowering	f = 70.44/(1+exp(-(x-4.58)/ 1.70))	0.92	4.5	4.2 - 4.9	0.45
